# Herbal medicine use in pregnancy: results of a multinational study

**DOI:** 10.1186/1472-6882-13-355

**Published:** 2013-12-12

**Authors:** Deborah A Kennedy, Angela Lupattelli, Gideon Koren, Hedvig Nordeng

**Affiliations:** 1 The Motherisk Program, Division of Clinical Pharmacology and Toxicology, Department of Pediatrics, The Hospital for Sick Children, University of Toronto, M5G 1X8 Ontario, Canada; 2 School of Pharmacy, University of Oslo, Oslo, Norway; 3 Division of Mental Health, Norwegian Institute of Public Health, Oslo, Norway

**Keywords:** Herbal medicine, CAM, Complementary and alternative medicine, Pregnancy, Information sources

## Abstract

**Background:**

The use of complementary and alternative medicines (CAM) is growing in the general population. Herbal medicines are used in all countries of the world and are included in the top CAM therapies used.

**Methods:**

A multinational study on how women treat disease and pregnancy-related health ailments was conducted between October 2011 and February 2012 in Europe, North and South America and Australia. In this study, the primary aim was to determine the prevalence of herbal medicine use in pregnancy and factors related to such use across participating countries and regions. The secondary aim was to investigate who recommended the use of herbal medication in pregnancy.

**Results:**

There were 9,459 women from 23 countries participating in the study. Of these, 28.9% reported the use of herbal medicines in pregnancy. Most herbal medicines were used for pregnancy-related health ailments such as cold and nausea. Ginger, cranberry, valerian and raspberry were the most commonly used herbs in pregnancy. The highest reported rate of herbal use medicines was in Russia (69%). Women from Eastern Europe (51.8%) and Australia (43.8%) were twice as likely to use an herbal medicine versus other regions. Women using herbal medicines were characteristically having their first child, non-smokers, using folic acid and consuming some alcohol in pregnancy. Also, women who were currently students and women with an education other than a high school degree were more likely to use herbal medicines than other women. Although 1 out of 5 women stated that a physician had recommended the herbal use, most women used herbal medicine in pregnancy on their own initiative.

**Conclusions:**

In this multinational study herbal medicine use in pregnancy was high although there were distinct differences in the herbs and users of herbal medicines across regions. Most commonly the women self-medicated with herbal medicine to treat pregnancy-related health ailments. More knowledge regarding the efficacy and safety of herbal medicines in pregnancy is warranted.

## Background

In the past two decades the use of complementary and alternative medicine (CAM) has grown considerably worldwide [1]. In the European Union, the prevalence of herbal medicine use ranges from 5.9% to 48.3%, whereas herbal medicine use in the USA and Canada is estimated to be 17.9% and 12%, respectively [2-4]. Herbal medicines are used in all countries of the world and are included in the top CAM therapies used [1,4-6]. Surveys on the use of herbal medicines in pregnancy have reported a wide range of herbal medicine use. In the Western world, prevalence estimates of herbal medicine use in pregnancy varies considerably across countries, ranging from 52-58% in Australia and the United Kingdom [7,8], to 40-48% in Norway and Italy [9,10] and 6-9% in Canada and the US [11,12]. It is difficult to ascertain whether the differences in prevalence are caused by differences in study design, methodology and exposure ascertainment across studies or whether they represent true differences in herbal medicine use. Uniform data collection simultaneously in different countries may overcome such drawbacks, allow for inter-country comparability and shed light on differences in the use of herbal medicines in various countries.

The top herbals medicines used in pregnancy have been found to include ginger, cranberry, raspberry, echinacea and chamomile, with geographical variations [13]. Often herbal medicines have been used as a complementary therapy, concurrent with pharmaceutical drugs rather than strictly as an alternative [9].

In general, studies have found that herbal medicine users are women over the age of 35, with a higher education and prior pregnancy [14,15]. A recent review summarized the reported motivations for a woman’s use of CAM therapies in pregnancy which include: the belief that these therapies provided safe alternatives to pharmaceutical drugs, an appreciation of a holistic potential afforded by these therapies and a desire to have control and satisfaction in their pregnancy experience [13].

Most of the previous surveys conducted in the use of herbal medicines in pregnancy have been performed in a specific antenatal clinic or a limited geographical area and have rarely explored the use of herbal medicines for chronic disease conditions. We sought to leverage the technological advances afforded by the internet to reach pregnant women in many countries simultaneously and explore the use of herbal medicines for both pregnancy related ailments and chronic conditions.

The objective of this study was to characterize the prevalence of herbal medicine use in pregnancy and the characteristics of herbal medicine users in pregnancy from a multinational perspective. Specifically, we sought to investigate the reasons for herbal medicine use and the herbal medicines used for both chronic and pregnancy-related health ailments. The secondary objective was to examine who recommended the use of herbal medicine to the pregnant women.

## Methods

This was a multinational, cross-sectional, internet-based study. Invitations to participate in the project were extended to countries of the European Network of Teratology Information Services (ENTIS), Mothersafe in Australia, the Organization of Teratology Information Specialists (OTIS) in North and South America and other European institutions conducting public health research. The following countries agreed to participate: Australia, Austria, Canada, Croatia, Finland, France, Iceland, Italy, Norway, Poland, Russia, Serbia, Slovenia, Sweden, Switzerland, The Netherlands, United Kingdom, and USA. Data originating from some South and Central America countries (Argentina, Bolivia, Brazil, Chile, Colombia, Ecuador, Paraguay, Peru, Uruguay and Venezuela) was also collected.

To minimize recall bias, women were eligible to participate if they were pregnant or had at least one child under one year of age. Study participants were categorized by reported country of residence at the time of the completion of the questionnaire. Country of residence was combined into six regions: Western Europe, Northern Europe, Eastern Europe, North America, South America and Australia.

An online self-completed questionnaire was developed and was open to the public via utilization of banners (invitations to participate in the study) on national websites and/or social networks commonly visited and consulted by pregnant women and/or new mothers. The websites also included Teratology Information Services (TISs), Organization for Teratology Information Services (OTIS) webpages, social networking sites, pregnancy forums and e-mail newsletters. Detailed information on recruitment locations and internet penetration rates are summarized in Additional file 1. The recruitment phase was between October 1 2011 and February 29 2012. The survey questionnaire was administered by Questback (http://www.questback.com). In each participating country, the questionnaire was accessible online for a period of two months.

The questionnaire was first developed in Norwegian and English. Translation into the relevant languages was performed; back-translation to English was done for specific parts of the questionnaire (i.e. psychometric scales) by two independent native speakers and/or translators. The questionnaire was translated into the following languages; Croatian, Dutch, Finnish, French, German, Icelandic, Italian, Norwegian, Polish, Russian, Serbian, Slovenian, Spanish and Swedish. A pilot phase of the study was carried out in September 2011 in Norway, Sweden, Finland and Italy (n = 47). The analysis of pilot data did not identify any major issues to the questionnaire. Collected data were scrutinized for the presence of potential duplicates (based on reported country of residency, socio-demographic characteristics, date and exact time of questionnaire completion) but none were identified. As a means of assessing external validity, socio-demographic and lifestyle characteristics of the questionnaire respondents was compared to those of the general birthing population in the corresponding country. National Statistics Bureau reports or national studies were used as the source for these comparisons (see Additional file 2).

Consent was obtained as follows: upon clicking on the link to the survey, each woman was presented with a description of the study and asked whether she was willing to participate. Informed consent was given by ticking a Yes response. Study approval was obtained from the Regional Ethic Committee, Region South-East in Norway and relevant Ethics Boards in each specific country. Permission to analyze the herbal medicine study data was also obtained from the Research Ethic Board of the Hospital for Sick Children, Toronto, Canada. All data were handled and stored anonymously.

The online questionnaire captured data on maternal health, socio-demographic, and lifestyle characteristics as well as use of herbal, and conventional medicines in pregnancy. The question “Did you take any herbal preparations during pregnancy (e.g. ginger, echinacea, valerian, cranberries)? If yes, please provide the name of all herbal preparations you have taken during pregnancy” was posed to all study participants. Women could report as free text entry the names of all herbal medicines used in pregnancy, along with the reason for its use, the period of use during pregnancy and the recommendation source(s). In addition, herbal medicine use could be reported under the specific questions about diseases and pregnancy-related health ailments. The relevant sections of the questionnaire are detailed in Additional file 3.

We defined herbal medicine according to the World Health Organization’s definition of any medicinal product based on herbs, herbal materials, herbal preparations and finished herbal products, that contain as active ingredients parts of plants, other plant materials, or combinations thereof [16]. Medicinal products based on animal components, vitamins, minerals or homeopathic products were not considered as herbal medicines.

The responses to the herbal medicine text field were coded according to a pre-determined classification list of herbs by the national coordinator in each participating country. The pre-determined classification list was compiled by the study development team and included the herb’s common name, latin name and a 7 character specific code following the format of the World Health Organization’s Anatomical Therapeutic Chemical (ATC) code convention as a means to standardize the coding in the questionnaire database. When a product name representing a multi-herbal combination or combination product was entered, an internet search on the product name was performed and the botanical ingredient(s) coded according to the pre-determined classification list. When the woman reported using several herbal medicines and several reasons for use consecutively, the order of reporting was used to match the herbal medicine and its use. The form of the herbal medicine was not specifically requested (tea, tablet, or tincture). Period of use was defined as weeks 1 - 12 (first trimester), 13-24 weeks (second trimester) and week 25 to delivery (third trimester) and presented as selection options. Several recommendation sources could be checked off: my own choice, family/friends, physician, midwife/nurse, pharmacist, herbal shop staff, internet, magazine/media/etc or other.

Reasons for use were recorded and grouped into the following categories: preparation for labor, health promotion, nausea, anemia, cold/flu, urinary tract infections (UTIs), pain conditions, constipation, heartburn, gastrointestinal disorders/flatulence, sedative/sleeping problems, restless legs, water retention and prevention of premature delivery.

Maternal characteristics included age, marital status, educational level, mother tongue, employment status, parity, pregnancy intention, and information on use of assisted reproductive technology. Life-style characteristics included folic acid use and smoking status before and during pregnancy and alcohol consumption after awareness of pregnancy. Both disease specific and questions regarding pregnancy-related health ailments and how they were treated were included in the questionnaire. Participating women were first presented with a list of questions related to acute/short-term illnesses (e.g. nausea, UTIs) and chronic/long-term disorders (e.g. asthma, depression) and asked whether they suffered from these conditions during pregnancy. In cases with an affirmative response, women were questioned about medication use for each individual indication as free-text entry fields. Conventional medications were coded according to the World Health Organization’s ATC classification system [17].

Descriptive statistics were used to calculate the prevalence (%) and associated standard error (SE) of herbal medicine use in pregnancy, reasons for use and information sources. If a point estimate had a SE greater than 50%, an “*” was used and the point estimate was not reported. Univariate and multivariate logistic regression analysis was used to identify significant factors associated with herbal medicine use among study participants. The analyzed maternal characteristics included region of residence, age, parity, marital status, employment status, education level, use of folic acid prior to and/or during pregnancy, alcohol use after awareness of pregnancy and smoking during pregnancy. First the univariate logistic regression model was fit for all explanatory variables. From this, the multivariate model was built and adjusted for relevant covariates. A p-value of <0.05 was considered statistically significant. The Hosmer and Lemeshow test was used to assess goodness of fit of the final multivariate model [18]. The data are presented as adjusted odds ratios (aOR) with 95% confidence intervals (CI). All statistical analysis was performed using the Statistical Package for the Social Sciences (SPSS) version 20.0 (IBM® SPSS® Statistics, Armonk, USA).

## Results

**Figure 1:**
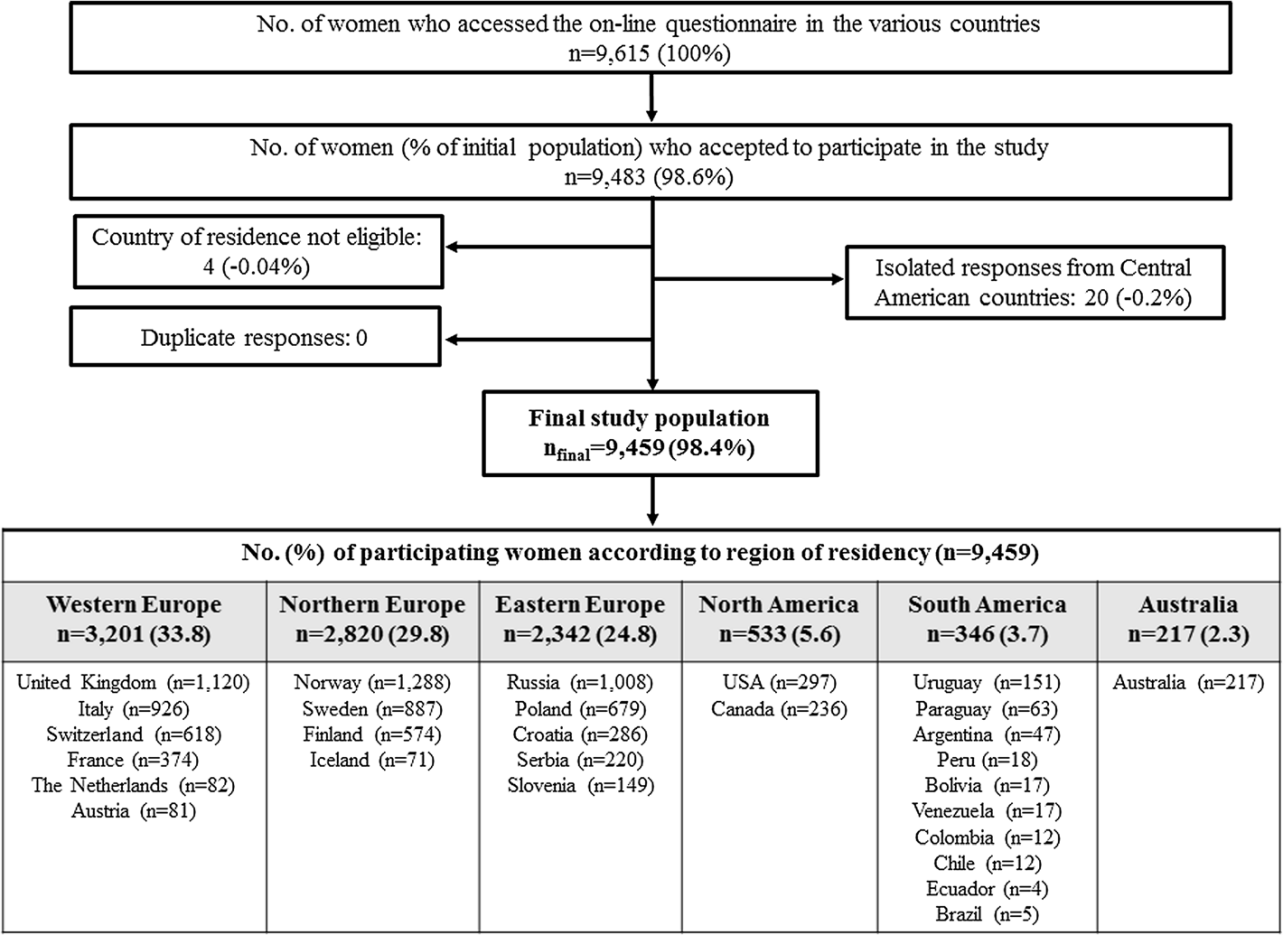
Participant flow-chart to achieve final analysis sample.

A total of 9,459 women responded to this internet survey from six different regions and 23 countries. The majority of study participants were women from Western Europe (n = 3,201), followed by Northern Europe (n = 2,820), Eastern Europe (n = 2,342), North America (n = 533), South America (n = 346) and Australia n = 217). Figure 1 summarizes the participant flow chart to achieve the final study population. The overall sample reflected well the birthing populations in each participating country with respect age, parity and smoking habits (see Additional file 2). However, our sample comprised a greater number of women with high educational levels versus the general birthing population in each country. Women in Australia, USA, The Netherlands, Slovenia, Croatia and Serbia were somewhat older than their respective countries’ birthing populations; while, women in Austria, Italy and Sweden were slightly more often primiparous.

Fifty-four percent (5,089/9,459; 53.8%) of respondents were pregnant at the time of completion of the questionnaire with the remainder (46.2%) having delivered their babies within the previous year. The mean gestational week was 22.4 among pregnant women. The majority of the new mothers (51.8%) had babies under 28 weeks of age.

### Prevalence of herbal medicine use in pregnancy

**Figure 2:**
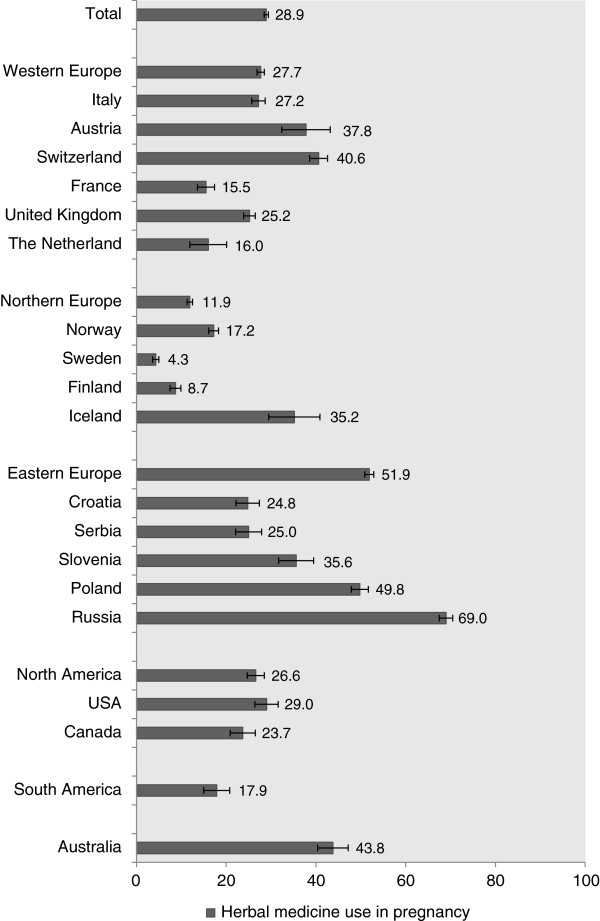
Percentages of women reporting the use of herbal medicine during pregnancy by region and country of residence.

The use of herbal medicines in pregnancy was reported by 2,735 out of the 9,459 responders (28.9%). Australia, Poland and Russia had the highest reported rates of herbal medicine users (Figure 2).

In total, 5,023 herbal medicines were reported by 2,735 women (overall average 1.6 herbal medicines each). The five most frequently used herbal medicines were ginger, cranberry, valerian, raspberry and chamomile. Table 1 summarizes the top twenty herbal medicines used overall and by region. These top twenty herbs represented over 70% of the herbs that were used in pregnancy with ginger and cranberry used by 46.2% of women using herbals. Overall, there were 134 different herbs used. Among the top twenty herbs, there was some regional variation in the use of specific herbal medicines. Motherwort, centaury and lovage were used only by women from Eastern European countries. Cowberry was only reported by women from Europe while, Uva ursi was used by women in Western and Eastern Europe only.

**Table 1:** The 20 most frequently used herbal medicines in pregnancy, overall and according to region

	**Overall use**		**REGION**						
**Top 20 herbal medicines**	**Total number of herb used (n)**	**% ± SE**	**Western Europe n = 888 % ± SE**	**Northern Europe n = 335 % ± SE**	**Eastern Europe n = 1,213 % ± SE**	**North America n = 142 % ± SE**	**South America n = 62 % ± SE**	**Australia n = 95 % ± SE**	**Total number of women n = 2,735 % ± SE**
Ginger	643	12.8 ± 0.5	28.5 ± 1.5	39.1 ± 2.7	12.2 ± 0.9	40.8 ± 4.1	4.8 ± 2.7	52.6 ± 5.1	23.5 ± 0.8
Cranberry	622	12.4 ± 0.5	10.6 ± 1.0	22.4 ± 2.3	35.7 ± 1.4	6.3 ± 2.0	*	10.5 ± 3.1	22.7 ± 0.8
Valerian	391	7.8 ± 0.5	6.9 ± 0.9	*	26.4 ± 1.3	2.8 ± 1.4	*	-	14.3 ± 0.7
Raspberry	301	6.0 ± 0.4	14.8 ± 1.2	11.0 ± 1.7	7.4 ± 0.8	17.6 ± 3.2	-	18.9 ± 4.0	11.0 ± 0.6
Chamomile	194	3.9 ± 0.3	4.2 ± 0.7	1.2 ± 0.6	10.5 ± 0.9	5.6 ± 1.9	27.4 ± 5.7	*	7.1 ± 0.5
Peppermint	188	3.7 ± 0.3	6.5 ± 0.8	2.1 ± 0.8	8.1 ± 0.9	8.5 ± 2.3	12.9 ± 4.3	5.3 ± 2.3	6.9 ± 0.5
Dog rose	149	3.0 ± 0.2	*	*	11.8 ± 0.9	*	*	-	5.4 ± 0.4
Cowberry	142	2.8 ± 0.2	*	*	11.5 ± 0.9	-	-	-	5.2 ± 0.4
Psyllium	132	2.6 ± 0.2	6.1 ± 0.8	1.8 ± 0.7	1.4 ± 0.3	19.7 ± 3.3	12.9 ± 4.3	20.0 ± 4.1	4.8 ± 0.4
Rosemary	98	2.0 ± 0.2	*	-	7.7 ± 0.8	-	-	*	3.6 ± 0.4
Centaury	94	1.9 ± 0.2	-	-	7.7 ± 0.8	-	-	-	3.4 ± 0.3
Lovage	94	1.9 ± 0.2	-	-	7.7 ± 0.8	-	-	-	3.4 ± 0.3
Lemon	93	1.9 ± 0.2	3.8 ± 0.6	*	4.0 ± 0.6	*	6.5 ± 3.1	3.2 ± 1.8	3.4 ± 0.3
Echinacea	92	1.8 ± 0.2	5.0 ± 0.7	4.8 ±1.2	1.5 ± 0.3	6.3 ± 2.0	*	3.2 ± 1.8	3.4 ± 0.3
Lemon Balm	84	1.7 ± 0.2	2.4 ± 0.5	*	4.9 ± 0.6	*	*	-	3.1 ± 0.3
Motherwort	79	1.6 ± 0.2	-	-	6.5 ± 0.7	-	-	-	2.9 ± 0.3
Garlic	78	1.6 ± 0.2	*	*	5.6 ± 0.7	2.8 ± 1.4	-	*	2.9 ± 0.3
Fiber crops	66	1.3 ± 0.2	3.6 ± 0.6	*	0.5 ± 0.2	9.9 ± 2.5	*	8.4 ± 2.8	2.4 ± 0.3
Uva ursi	65	1.3 ± 0.2	0.1 ± 0.1	-	5.3 ± 0.6	-	-	-	2.4 ± 0.3
**Total**	**3,605**	**72.0** ± 0.6							
**Summary of herbal medicine used**									
Total no. of herbs used			1,261	379	2,904	233	110	136	5,023
No. of multiherb products			98	10	368	8	0	1	485
Average no. of herbs used			1.4	0.6	1.1	1.4	1.4	1.4	1.6
No. of different herbs			90	54	83	42	38	24	134

SE: Standard error. Standard errors were calculated for all percentages; however, where the SE > 50% the point estimate is not reported and an “*” is used.

Nine percent of the herbal users indicated that they had a chronic illness; however, less than one percent of these women used an herbal medicine to manage their chronic illness (data not shown).

Additional file 4 summarizes the ten most common reasons for using herbal medicines and the most frequently used herbs, overall and by region. Common concerns of pregnancy such as cold/flu, nausea, sleeping problems, constipation and labor preparation were among the top reasons for using herbal medicines. There were similarities in the most commonly used herbs used for these ailments across regions. In addition, UTIs in pregnancy were commonly treated with cranberry.

### Factors associated with herbal medicine use

Additional file 5 summarizes the overall and regional maternal factors related to herbal medicine use in pregnancy. Region of residence was a significant factor in herbal medicine use. Compared to women in Western Europe, women in Australia and Eastern Europe were twice as likely to use an herbal medicine in pregnancy, while women from Northern Europe were significantly less likely to use an herbal medicine. Women using herbal medicines were characteristically having their first child, non-smokers, using folic acid and consuming some alcohol in pregnancy. Also, women who were currently students and women with an education other than a high school degree were more likely to use herbal medicines than other women.

### Sources of the recommendation to use herbal medicines

Table 2 summarizes the sources for women’s use of an herbal medicine, by region (country details in Additional file 6). In most cases the women used herbal medicine on their own initiative. Informal sources (family & friends, internet, magazines and media) represent over 30% of sources that women indicated that they accessed. The second highest source was physicians. In the Eastern European countries, a physician’s recommendation was the most frequently indicated source.

**Table 2:** Source of the recommendation to use herbal medicine in pregnancy according to region

	**Percentage of women indicating each recommendation source**									
	**Total responses**	**Own initiative**	**Physician**	**Family & friends**	**Internet**	**Midwife, nurse**	**Pharmacy personnel**	**Magazine or media**	**Herbal shop**	Other**
	**N**	**% ± SE**	**% ± SE**	**% ± SE**	**% ± SE**	**% ± SE**	**% ± SE**	**% ± SE**	**% ± SE**	**% ± SE**
**Total**	**3,961**	**28.6 ± 0.7**	**21.6 ± 0.7**	**16.8 ± 0.6**	**11.3 ± 0.5**	**7.8 ± 0.4**	**6.1 ± 0.4**	**3.3 ± 0.3**	**3.0 ± 0.3**	**1.5 ± 0.2**
Western Europe	1,315	27.6 ± 1.2	14.7 ± 1.0	18.6 ± 1.1	11.3 ± 0.9	10.2 ± 0.8	7.7 ± 0.7	4.0 ± 0.5	4.0 ± 0.5	2.1 ± 0.4
Northern Europe	548	31.9 ± 2.0	8.6 ± 1.2	19.2 ± 1.7	15.7 ± 1.6	9.1 ± 1.2	5.3 ± 1.0	3.6 ± 0.8	5.7 ± 1.0	0.9 ± 0.4
Eastern Europe	657	28.0 ± 1.8	34.5 ± 1.9	13.3 ±1.3	10.3 ± 1.2	4.9 ± 0.8	5.6 ± 0.9	2.6 ± 0.6	0.8 ± 0.3	*
North America	209	31.6 ± 3.2	8.6 ± 1.9	18.2 ± 2.7	11.5 ± 2.2	13.9 ± 2.4	2.4 ± 1.1	3.3 ± 1.2	3.8 ± 1.3	*
South America	71	22.5 ± 5.0	12.7 ± 4.0	36.6 ± 5.7	8.5 ± 3.3	*	*	5.6 ± 2.7	9.9 ± 3.5	-
Australia	161	29.8 ± 3.6	9.9 ± 2.4	21.1 ± 3.2	9.3 ± 2.3	9.3 ± 2.3	8.1 ± 2.2	*	5.6 ± 1.8	*

SE: Standard error. Standard errors were calculated for all percentages; however, where the SE > 50% the point estimate is not reported and an “*” is used.

Note: Women were permitted to indicate more than one source for the recommendation to take an herbal medicine. **Includes sources such as prenatal yoga class or CAM practitioner.

## Discussion

This is the first multinational study of the use of herbal medicine by women during pregnancy and provides insight into the use of these products in several countries, in some, for the first time. Three findings are specifically important. Firstly, we found that the use of herbal medicine in pregnancy varied considerably between countries, but that many of the same herbs are used. Secondly, there were no specific features that characterised the woman who used herbal medicines in pregnancy across all countries. Thirdly, in most countries women relied on informal information sources in their decision to use an herbal medicine in pregnancy.

The use of herbal medicines, overall, was 28.9%, and ranged from a low of 4.3% in Sweden to 69% in Russia. This range is consistent with results in other studies [9,10,13,19-23]. The previously reported rate of herbal use in Finland was 3.6%; 1/3 of what we find in our study. We found lower prevalence rates of herbal medicine use in Norway, the UK and Italy than previously reported [9,10,14], but higher rates in Finland and Sweden [15,24]. In Australia, previous studies have a reported prevalence of the use of herbal medicine of between 11% to 56%, which is consistent with our results [25-27]. This variability may reflect differences in data collection or differences in time trends. The broad availability of the study questionnaire might, in fact, promote a more representative study population rather than reflecting just an antenatal clinic or specific geographical area. The highest prevalence rate of the use of herbal medicines in pregnancy in Russia, coupled with the 38% of Russian women indicating that the recommendation to use an herbal medicine came from a physician could, in part, be due to the acceptance of the use of herbal medicines by Russian physicians. A 2008 survey of physicians in Russia found that 76% reported the use of phytotherapy and 71% of herbal medicines in their practice [28]. This country also was found to have the lowest allopathic medication use in the survey (Lupattelli A *et al*: Medication use in pregnancy: a multinational perspective. 2013 (submitted)).

The top herbal medicines used have been previously reported to include ginger, cranberry, raspberry, chamomile, valerian, and echinacea. This is consistent with the present survey as well. The use of these herbals was primarily for ailments related to pregnancy; nausea, UTIs and preparation for labor, rather than to assist with chronic diseases. Ginger was not only used for nausea but for cold and flu’s, perhaps due to its diaphoretic properties [29], health promotion and gastrointestinal disorders. A literature review the effect of ginger on nausea and vomiting in pregnancy found that ginger maybe helpful; however, the study results were inconsistent [30]. The results of a large cohort study with 1,020 exposed pregnancies demonstrated that there was no increase in congenital malformations or poor pregnancy outcomes after the use of ginger during pregnancy [31].

Cranberry was used for multiple purposes as well; cold and flu’s, UTIs, health promotion and water retention. Cranberry’s effectiveness in UTI prophylaxis and UTI treatment has not been demonstrated [32]. Taking into account the fact that untreated UTIs can increase the risk of pregnancy complications, pyelonephritis, impacting fetal growth and preterm delivery [33], the high use of herbals for UTI in our study is of concern. Health care professionals should inform pregnant women to use antibiotics and not herbals to treat UTI in pregnancy.

Raspberry was used for colds and flu’s and health promotion, where its properties would likely provide little benefit, in addition to the usually associated indication as a uterine tonic in preparation for labor [34]. A recent literature review concluded that there was lack of evidence for safety and efficacy in promoting effective labor and questioned its use in pregnancy in light of the weak evidence that currently exists [35].

Many sources have suggested that women who use CAM medicine are characterized as being between the ages of 31-40 years, having higher education and income levels and used CAM in a previous pregnancy [7,13]. Overall, the study participants who used herbal medicines were having their first child, more often students and less likely to work as healthcare providers, with an educational level other than high school, non-smokers, using both folic acid and alcohol during pregnancy. Several of the factors associated with herbal medicine users are different from previously reported studies in terms of age and education [13]. However, Forster *et al*. did find in their study that herbal medicine users were more likely to be nulliparous [27]. These differences from previous studies may simply reflect a more representative user group that we were able to reach via the use of the internet rather than being limited to a specific antenatal clinic or geographical area. Overall, maternal age was not a significant determinant of herbal use during pregnancy apart from Western and Eastern Europe. In the former region use of herbal remedies was less prevalent among women younger than 20 years of age than the 21-30 year counterpart, whereas in the latter it was less prevalent among women of 31-40 years of age and more common among younger women (less than 20 years). Across the regions, there were also differences in the characteristics of herbal users with respect to parity and employment status. In fact, while parity and employment status were not significant determinants of herbal use during pregnancy in either North or South America or Australia, they were so in both Western and Eastern Europe. One consistent characteristic across Europe and North America, which has not been reported previously, is that herbal users were more likely to continue to consume alcohol once they were aware they were pregnant. This finding might reflect a certain open or extravert life style where both herbal medicine and alcohol consumption use is common [36]. Given that alcohol is a known teratogen and there is no known safe amount to consume when pregnant, this would to seem to contradict the objective of using substances that are perceived to be safe that one might associate with the use of herbal medicines [37].

Informal information sources, such as one’s own initiative, friends/family, were the primary sources that women indicated as involved in their decision to use an herbal medicine in pregnancy. As several authors have suggested, this may well reflect a woman’s desire to have a natural approach to pregnancy [7,38], or perhaps these women feel more active in their health and feel more comfortable making their own decisions or a desire to use less conventional medication. This use could represent a concern, as studies have found that women will often not communicate the use of herbal medicines to their health care providers [14].

There are several strengths in this study. The use of an internet-based survey permitted access by a large number of women regarding their use of herbal medicine in pregnancy and provided insights on its use in regions and countries that have not previously been reported. A comparison of the survey population to the participating countries’ birthing populations found that the responding population were of similar age, parity and smoking habits, but had a higher education level. This may suggest that herbal medicine users may be overrepresented in this survey since higher education levels have been associated with herbal medicine use and higher education was a significant determinant of herbal use in several regions in the survey results. Similarly, internet users cluster in higher socioeconomic classes. The regional variations in the age of herbals users, suggesting that herbal use is higher amongst younger, nulliparous women may reflect an emerging trend in some countries.

There are also several limitations that should be mentioned. Firstly, as an internet based study, this may introduce a population selection bias by access to the internet. Internet penetration rates are high among the target population in many of the participating countries, and recent epidemiological studies indicate reasonable validity of web-based recruitment methods [39-41]. In Europe, the penetration rates range from about 50% in Russia and Serbia, to 100% in Iceland [41,42]. In the USA, Canada and Australia approximately 80-90% of the population has access to the internet, though lower rates (about 50%) apply to South America [42-45]. Hence, the degree to which our findings can be extrapolated to the target population is based on the representativeness of the respondents to the general birthing populations in each country.

Secondly, a conventional response rate could not be calculated because of the utilization of multiple websites in each participating country. However, of the women who expressed their willingness to participate or not in the study, 98.6% took part and completed the online questionnaire. Thirdly, the potential for recall bias cannot be excluded here since we were reliant upon women to recall which herbal medicines were taken and, for 1,351 women (49.3%), the events were up to one year in the past. Also, the duration of use of the herbal medicine in pregnancy was not captured in the survey. However, as 75% of the herbals users used herbal medicines for pregnancy-related health ailments, we assume that short term use was most common. There may also be under reporting of some herbal medicines as herbal names were not specifically queried in the questionnaire. Further, when a multi-herbal product was provided as a response, an internet search for its ingredients was performed. It is possible that the composition of named products could vary from country to country and these differences would not have been captured. Our results must be interpreted with these strengths and limitations in mind.

## Conclusions

In this multinational study use of herbal medicine in pregnancy was high. In total, 134 different herbs were used, most frequently ginger, cranberry, valerian and raspberry for pregnancy-related health ailments. There was variability in both the prevalence of the use and users of herbal medicines in pregnancy across regions. Many women primarily used informal information sources in their decision to use an herbal medicine in pregnancy in most regions; however, in the Eastern European countries, physicians’ recommendations were cited most often.

## Competing interests

The authors have no conflicts of interest to declare. Gideon Koren holds the Research Leadership for Better Pharmacotherapy During Pregnancy and Breastfeeding (Sickkids Hospital) and the Ivey Chair in Molecular Toxicology (University of Western Ontario).

## Authors’ contributions

HN and AL conceived the idea for the study and participated in its design and coordination. DAK analyzed the data and drafted the manuscript. AL participated in the data analysis. GK, AL, and HN critically reviewed the manuscript and contributed intellectual content. All authors read and approved the final manuscript.

## Pre-publication history

The pre-publication history for this paper can be accessed here:

http://www.biomedcentral.com/1472-6882/13/355/prepub

## Supplementary Material

Additional file 1 **Websites used for recruitment and internet penetration rates in each participating country.** A table which summarizes the websites where the questionnaire was available in each country and the associated internet penetration rates. Click here for file

Additional file 2 **Socio-demographic characteristics of the study population and general birthing population on individual country.** A table comparing the study population with the general birthing population with respect to maternal age at delivery, smoking status. This information was used to determine the representativeness of the study population. Click here for file

Additional file 3 **Medication use in pregnancy with focus on attitudes and perception of risk.** This document contains the relevant sections of the questionnaire. Click here for file

Additional file 4 **The ten most common reasons for using herbal medicines and the most frequently used herbs.** A table summarizing the most common reasons given for using herbal medicines and associated most frequently used herb(s), overall and by region. Click here for file

Additional file 5 **Factors associated with herbal medicine use, overall and by region.** A table summarizing the maternal socio-demographic and lifestyle characteristics of herbal users in this study. This information is presented overall and by region. Click here for file

Additional file 6 **Source of the recommendation to use herbal medicine in pregnancy, by region and country.** A table summarizing the information sources for using herbal medicine in pregnancy, by region and country. Expanded details from Table 2. Click here for file
