# Supplementary material for: Herbal medicine use in pregnancy: results of a multinational study
Source: BMC Complement Altern Med. 2013 Dec 12;13:355. doi: 10.1186/1472-6882-13-355 (PMC4029224; doi:10.1186/1472-6882-13-355)
Supplement: Additional file 1 — Websites used for recruitment and internet penetration rates in each participating country. A table which summarizes the websites where the questionnaire was available in each country and the associated internet penetration rates. [file 1472-6882-13-355-S1.pdf]

Additional file 1: Websites used for recruitment and internet penetration rates in each participating country

Country	***Website used for recruitment***	***Internet penetration rates****
	Western Europe	
United Kingdom	*Targeted email to pregnancy forum subscribers*: www.bounty.com<br><br>*Pregnancy Forums*: www.pregnancyforum.co.uk; www.pregnancyforum.org.uk	93% [1]
Italy	*Pregnancy Forums*: www.gravidanzaonline.it; www.forumsalute.it; www.mammole.it; www.pianetamamma.it; www.miobambino.it<br><br>*Targeted email to pregnancy forum subscribers*: www.gravidanzaonline.it	70% [1]
Switzerland	www.bebe-bebe.com; www.swissmom.ch	84.2% [2]
France	www.aufeminin.com (*Including ipad application to website subscribers*)	91% [1]
The Netherlands	www.lareb.nl; www.gezondzwangerzijn.nl; www.babybytes.nl	98% [1]
Austria	www.schwangerschaft.at; www.schwangerschafts-blog.at; www.fratz.at; www.netdoctor.at; www.babycenter.at; www.baby-boom.at; www.ekiz-	93% [1]

Country	*Website used for recruitment*	*Internet penetration rates*[*]
	dachverband.at; www.babyguide.at	
	Northern Europe	
Norway	www.barnimagen.com; www.klikk.no; www.jormorsiri.no; www.tryggmamamedisin.no	99% [1]
Sweden	www.barntotal.se; www.minbebis.com; www.se.babycenter.com; www.socmed.gu.se	99% [1]
Finland	www.vauva.fi; www.meidanperhe.fi; www.kaksplus.fi	99% [1]
Iceland	*Pregnancy Forums*: www.bland.is	100% [1]
	Eastern Europe	
Russia	www.babyblog.ru; www.littleone.ru<br><br>*Pregnancy Forums*: www.woman.ru; www.9months.ru; www.bemam; www.280dney.ru; www.iampregnant.ru www.pregnancy.org.ua; www.baby.ru; www.mama66.ru; www.spuzom.ru	47.7% [2]
Poland	www.zzief.umlub.pl<br><br>*Pregnancy Forums*: www.ebrzuszek.pl; www.babyboom.pl; www.zapytajpolozna.pl; www.planujemydziecko.pl; www.twoja-ciaza.com.pl	84% [1]
Croatia	www.cybermed.hr	80% [1] (data

Country	*Website used for recruitment*	*Internet penetration rates*[*]
		from 2010)
Serbia	www.ringeraja.rs	52% [1](data from 2009)
Slovenia	*Pregnancy Forums*: www.med.over.net	92% [1]

[*]Indicates the frequency of internet access - at least once a week, including every day - among individuals aged 25-34 years. Differences between men and women were relatively small. Slightly more than two thirds of men (70%) and 65% of women used the Internet regularly.

Country	*Website used for recruitment*	*Internet penetration rates*
North America		
USA/Canada	www.otispregnancy.org; Facebook page of OTIS; www.babyontheway.com.ca; www.justmommies.com<br><br>*Pregnancy Forums*: www.babyandbump.com www.babycentre.com.ca; www.thecradle.com; www.talk.sheknows.com; www.parenting.com	Canada: 94% [3] [**]<br>USA: 80.2% [4][§]
South America	www.otispregnancy.org; Facebook page of OTIS<br><br>*Pregnancy Forums*: www.semanaasemana.com;	South America: 48.2% [2]

	www.univision.com; www.elembarazo.net	

** Indicates individuals aged 16-45 years who used the internet for personal use.

§Indicates individuals > 18 years old, access from anywhere; household internet for women is equal to 68.1%; higher percentages are observed for people aged 25-54 years.

Country	***Website used for recruitment***	***Internet penetration rates§§***
Australia	www.mothersafe.org.au; www.bubhub.com.au<br><br>*Pregnancy Forums*: www.abds.org.au; www.birth.com.au	83% [5]

§§Indicates households with access to the internet at home.

References

1. Seybert H: **Internet use in households and by individuals in 2011.**, vol. 66: Eurostat. Statistics in focus. 2011.
2. **Internet World Stats. Usage and population statistics** [http://www.internetworldstats.com/]. Last accessed: November 13, 2012.
3. **Individual Internet use and E-commerce (2010)** [http://www.statcan.gc.ca/daily-quotidien/111012/dq111012a-eng.htm]. Last accessed: November 13, 2012.
4. **United States Census Bureau. The 2012 Statistical Abstract. Information & Communications: Internet Publishing and Broadcasting and Internet Usage.** [http://www.census.gov/compendia/statab/cats/information_communications/internet_pu

blishing_and_broadcasting_and_internet_usage.html.]. Last accessed: November 13, 2012.

5. **Australian Bureau of Statistics. Household Use of Information Technology, Australia, 2010-11.** [http://www.abs.gov.au/ausstats/abs@.nsf/Latestproducts/8146.0Main%20Features12010-11?opendocument&tabname=Summary&prodno=8146.0&issue=2010-11&num=&view=]. Last accessed: November 13, 2012.
