# Supplementary material for: Herbal medicine use in pregnancy: results of a multinational study
Source: BMC Complement Altern Med. 2013 Dec 12;13:355. doi: 10.1186/1472-6882-13-355 (PMC4029224; doi:10.1186/1472-6882-13-355)
Supplement: Additional file 2 — Socio-demographic characteristics of the study population and general birthing population on individual country. A table comparing the study population with the general birthing population with respect to maternal age at delivery, smoking status. This information was used to determine the representativeness of the study population. [file 1472-6882-13-355-S2.pdf]

Additional file 2: Socio-demographic characteristics of the study population and general birthing population on individual country

Table 2a: Socio-demographic characteristics in Western European countries (United Kingdom (UK), Italy and Switzerland)

	**Study sample in the UK n=1,120**	**General birthing population in UK* LB=723,165** [1]	**Study sample in Italy n=926**	**General birthing population in Italy LB=546,606** [2]	**Study sample in Switzerland n=618**	**General birthing population in Switzerland LB=80,808** [2]
	**(%)**	**(%)**	**(%)**	**(%)**	**(%)**	**(%)**
**Mean Age +/- sd**	30.5 +/- 5.2	29.6 [1]	32.3 +/- 5.0	31.3 [3]	31.6 +/- 4.3	31.4 [4]
Marital status						
In marriage	63.3	53.2 [1]	68.8	75.1 [2]	80.0	80.7 [4]
Outside marriage	36.7	46.8 [1]	31.2	31.5 [2]	20.0	19.3 [4]
Parity						
No previous children	48.0†	41.9 [1] †	59.7	48.7 [5]	53.2	-
Educational level						
Less than high school	0.6	16.5 [1]	7.0	25.2 [6]	11.0	11.7 [6]
High school	27.9	37.2 [1]	47.2	49.2 [6]	13.6	49.2 [6]
More than high school	52.1	46.3 [1]	44.3	25.6 [6]	47.2	39.1 [6]
Other	19.3	-	1.5	-	28.2	-
Women smoking before pregnancy	25.2	25.7 [7]	34.2	33.3 [3]	25.1	25.4 [7]
Women smoking during pregnancy	7.1‡	13.2 [8] ‡	10.5	22.7 [9]	5.5	6.6 [10]
Use of alcohol during pregnancy	28.3	24.0 [11] §	17.9	17.7 [9]	20.7	29.9 [10]
No. of respondents/No. live births **	0.9%		1.0%		4.6%	

Abbreviations: LB: Number of live births per year.

*The figures shown here are statistic estimates for England and Wales. Scotland and Northern Ireland have separate statistical reports. Since more than 85% of the study population in UK were resident in England and about 8% in Wales, we are only showing national statistic data for these two parts of the UK.

†Among married women only – as provided by the Statistics Bureau in the UK.

[‡]Among women resident in England only (as provided by the Statistics Bureau in the UK, data on 4th Quarter of 2011).
[§]Women reporting at least one occasion during pregnancy of consuming more than four drinks in a day.
[**]The ratio "No. of respondents/No. live births" is calculated as the proportion (%) of pregnancies included in the study among live births in the country in two months (period of data collection).

Table 2b: Socio-demographic characteristics in Western European countries (France, The Netherlands and Austria)

	Study sample in France n=374	General birthing population in France LB=824,263 [2]	Study sample in Netherlands n=81	General birthing population in Netherlands LB=180,060 [2]	Study sample in Austria n=82	General birthing population in Austria LB=78,109 [2]
	(%)	(%)	(%)	(%)	(%)	(%)
**Mean Age +/- sd**	29.6 +/- 4.9	30.1 [12]	32.0 +/- 6.4	31.0 [13]	30.6 +/- 4.6	30.0 [14]
Marital status						
In marriage	48.9	45.0 [2]	69.1	58.2 [13]	48.8	59.6 [15]
Outside marriage	51.1	55.0 [2]	30.8	41.8 [13]	51.2	40.4 [15]
Parity						
No previous children	52.9	44.2[16]	38.3	46.4 [13]	63.4	47.9 [15]
Educational level						
Less than high school	1.6	15.4 [6]	9.9	15.9 [6]	9.8	13.3 [6]
High-school	25.1	37.4 [6]	66.7	40.2 [6]	32.9	64.1 [6]
More than high school	57.0	47.2 [6]	23.5	43.9 [6]	40.2	22.7 [6]
Other	16.3	-	-	-	17.1	-
Women smoking before pregnancy	39.3	39.0 [16]	34.6	29.5 [7]	31.7	32.1 [17]
Women smoking during pregnancy	14.2	28.0 [16]	14.8	17.1 [18]	4.9	-
Use of alcohol during pregnancy	11.5	52.0 [19]	11.1	16-35 [13]	13.4	-
No. of respondents/No. live births *	0.3%		0.3%		0.6%	

Abbreviations: LB: Number of live births per year.

*The ratio "No. of respondents/No. live births" is calculated as the proportion (%) of pregnancies included in the study among live births in the country in two months (period of data collection).

Table 2c: Socio-demographic characteristics in Northern European countries (Norway, Sweden and Finland)

	**Study sample in Norway** **n=1,228**	**General birthing population in Norway LB=60,220** [2]	**Study sample in Sweden** **n=887**	**General birthing population in Sweden LB=111,770** [2]	**Study sample in Finland** **n=574**	**General birthing population in Finland LB=59,961** [2]
	**(%)**	**(%)**	**(%)**	**(%)**	**(%)**	**(%)**
Mean Age +/- sd	29.0 +/- 4.6	29.8 +/- 5.3 [20]	29.8 +/- 5.3	30.3 [21]	29.0+/-5.1	30.1 [22]
Marital status						
In marriage	39.1	46.0 [20]	40.7	45.8 [2]	59.4	57.8 [22]
Outside marriage	60.9	53.4 [20]	59.3	54.2 [2]	40.6	42.0 [22]
Unknown	-	0.6 [20]			-	0.2 [22]
Parity						
No previous children	41.4	42.4 [20]	63.1	44.9 [21]	35.5	42.2 [22]
Educational level		-				
Less than high school	4.5	14.7 [6]	5.2	11.1 [6]	8.2	7.1 [6]
High-school	28.0	31.4 [6]	30.0	38.2 [6]	36.4	44.5 [6]
More than high school	46.9	53.9 [6]	60.6	50.6 [6]	52.6	48.4 [6]
Other	20.7	-	4.2	-	2.8	-
Women smoking before pregnancy	33.5	36.5 [7]	25.0	27.2 [7]	36.7	19.7 [7]
Women smoking during pregnancy	6.8	7.0 [20]	5.4	6.5 [21]	11.7	15.2 [22]
Use of alcohol during pregnancy	4.1	7.4 [23]	7.2	5.9 [24]	13.9	-
No. of respondents/No. live births *	12.2%		4.8%		5.7%	

Abbreviations: LB: Number of live births per year.

*The ratio “No. of respondents/No. live births” is calculated as the proportion (%) of pregnancies included in the study among live births in the country in two months (period of data collection).

Table 2d: Socio-demographic characteristics in Northern European countries (Iceland)

	**Study sample in Iceland n=70 (%)**	**General birthing population in Iceland LB=4,492** [2] **(%)**
Age range (years)		
15-20	11.3	5.1 [25]
21-25	16.9	19.3 [25]
26-30	42.3	34.2 [25]
31-35	15.5	27.3 [25]
36-40	12.7	11.7 [25]
≥41	1.4	2.4 [25]
Marital status		
In marriage	31.0	35.0 [25]
Outside marriage	69.0	65.0 [25]
Parity		
No previous children	47.9	38.1 [25]
Educational level		
Less than high school	25.4	21.4 [6]
High-school	18.3	30.5 [6]
More than high school	43.7	48.1 [6]
Other	12.7	-
Women smoking before pregnancy	40.8	35.5 [7]
No. of respondents/No. live births *	9.3%	

Abbreviations: LB: Number of live births per year.

*The ratio "No. of respondents/No. live births" is calculated as the proportion (%) of pregnancies included in the study among live births in the country in two months (period of data collection).

Table 2e: Socio-demographic characteristics in Eastern European countries (Russia and Poland)

	Study sample in Russia n=1,008	General birthing population in Russia LB=1,796,629 [2]	Study sample in Poland n=679	General birthing population in Poland LB=388,416 [2]
	(%)	(%)	(%)	(%)
Mean Age +/- sd	27.7 +/- 4.8	27.4 [26]	27.1 +/- 4.1	28.6 [27] *
Marital status				
In marriage	85.3	73.9 [26]	85.0	79.4 [27]
Outside marriage	14.7	26.1 [26]	15.0	20.6 [27]
Parity				
No previous children	57.9	-	40.6	50.1 [27]
Educational level				
Less than high school	1.6	-	1.9	8.7 [27]
High-school	9.3	-	31.1	49.6 [27]
More than high school	75.1	-	65.1	41.6 [27]
Other	14.0	-	1.9	-
Women smoking before pregnancy	46.1	30.8 [28]	49.2	25.0 [29]
Women smoking during pregnancy	9.6	4.3-6.5 [30, 31]	12.8	22-30 [29]
Use of alcohol during pregnancy	26.0	60.0 [32]	9.6	15.3 [33]
No. of respondents/No. live births †	0.3%		1.0%	

Abbreviations: LB: Number of live births per year.

*Median age of women at birth, not mean age.

†The ratio "No. of respondents/No. live births" is calculated as the proportion (%) of pregnancies included in the study among live births in the country in two months (period of data collection).

Table 2f: Socio-demographic characteristics in Eastern European countries (Croatia, Serbia and Slovenia)

	**Study sample in Croatia n=286**	**General birthing population in Croatia LB=41,197** [2]	**Study sample in Serbia n=220**	**General birthing population in Serbia LB=65,598** [2]	**Study sample in Slovenia n=149**	**General birthing population in Slovenia LB=21,947** [2]
	**n (%)**	**(%)**	**n (%)**	**(%)**	**n (%)**	**(%)**
Mean Age +/- sd	29.1 +/- 4.5*	27.7 [34]	29.2 +/- 3.9*	28.7 [35]	31.7 +/- 4.5	30.4 [36]
Marital status						
In marriage	83.9	86.7 [34]	90.1	76.1 [35]	47.0	43.2 [36]
Outside marriage	16.1	13.3 [34]	9.9	23.9 [35]	53.0	56.8 [36]
Unknown	-	-	-	-	-	-
Parity						
No previous children	50.7	46.9 [34]	46.8	51.1 [35]	45.6	48.5 [36]
Educational level						
Less than high school	1.0	3.1 [34]	0.9	15.9 [37]	2.0	8.5 [36]
High-school	36.7	52.5 [34]	33.6	54.9 [37]	24.8	48.5 [36]
More than high school	61.2	44.4 [34]	61.8	29.2 [37]	69.1	43.0 [36]
Other	1.0	-	3.6	-	4.0	-
Women smoking before pregnancy	50.0	34.4 [34]	49.1	29.9 [38]	32.9	34.4 [7]
Women smoking during pregnancy	18.8	23.1 [39]	18.2	18.4 [40]	6.7	9.6-11.2 [41]
Use of alcohol during pregnancy	12.6	15.5 [42]	15.0	-	32.2	-
No. of respondents/No. live births †	4.2%		2.0%		4.1%	

Abbreviations: LB: Number of live births per year.
*Mean age for first child (as it is available from the Statistics Bureau reports in Croatia and Serbia).
†The ratio "No. of respondents/No. live births" is calculated as the proportion (%) of pregnancies included in the study among live births in the country in two months (period of data collection).

Table 2g: Socio-demographic characteristics in North American countries (USA, Canada) and Australia.

	Study sample in The USA n=297	General birthing population in USA LB=3,999,386 [43]	Study sample in Canada n=236	General birthing population in Canada LB=377,636 [44]	Study sample in Australia n=217	General birthing population in Australia LB=301,617 [45]
	**n (%)**	**(%)**	**n (%)**	**(%)**	**n (%)**	**(%)**
Age range (years)						
15-19	4.7	9.3 [43]	2.1	3.9 [44]	2.3	3.8 [45]
20-24	18.2	23.8 [43]	25.0	14.6 [44]	8.8	13.8 [45]
25-29	28.3	28.3 [43]	30.1	30.2 [44]	31.8	27.9 [45]
30-34	29.3	24.1 [43]	30.5	32.2 [44]	27.6	31.7 [45]
35-39	15.2	11.6 [43]	11.0	15.6 [44]	22.1	18.4 [45]
40-44	4.0	2.7 [43]	1.3	3.1 [44]	6.9	4.0 [45]
≥45	0.3	0.2 [43]	-	0.2 [44]	0.5	0.2 [45]
Mean Age +/- sd	29.3 +/- 6.1	-	28.3 +/- 5.2	29.6 [44]	31.1 +/- 5.7	30.7 [45]
Marital status						
In marriage	67.0	59.2 [46]	42.4	60.4 [44]	70.5	65.8 [45]
Outside marriage	33.0	39.9 [46]	57.6	28.8 [44]	29.5	34.2 [45]
Unknown	-	0.9 [46]	-	10.8[44]	-	-
Parity						
No previous children	41.1	40.1 [43]	48.3	43.3 [44]	47.9	43.8 [45]
Educational level						
Less than high school	2.7	17.4 [47]	1.3	8.4 [48]	0.5	20.6 [49]
High-school	25.3	24.4 [47]	24.6	-	29.0	
More than high school	62.0	58.2 [47]	67.8	69.6 [48]	63.1	56.0 [50]

	Study sample in The USA n=297	General birthing population in USA LB=3,999,386 [43]	Study sample in Canada n=236	General birthing population in Canada LB=377,636 [44]	Study sample in Australia n=217	General birthing population in Australia LB=301,617 [45]
	**n (%)**	**(%)**	**n (%)**	**(%)**	**n (%)**	**(%)**
Other	10.1	-	6.4	-	7.4	-
Women smoking before pregnancy	28.3	21.5 [51]	29.2	22.0 [52]	29.1	29.9 [53]
Women smoking during pregnancy	8.1	10.2 [54]	16.1	13.4 [48]	14.3	14.5 [55]
Use of alcohol during pregnancy	17.5	15.5 [51]	16.1	10.5 [48]	27.2	29.0 [56]
No. of respondents/No. live births*	0.04%		0.4%		0.4%	

Abbreviations: LB: Number of live births per year.
*The ratio "No. of respondents/No. live births" is calculated as the proportion (%) of pregnancies included in the study among live births in the country in two months (period of data collection).

References

1. **UK National Statistics. Characteristics of Birth 1 and 2/of mother 1 and 2, England and Wales. 2010** [http://www.statistics.gov.uk/hub/population/births-and-fertility/maternities/index.html]. Last accessed: November 12, 2012.
2. **Eurostat. Live births by mother's age at last birthday and legal marital status** [http://appsso.eurostat.ec.europa.eu/nui/show.do?dataset=demo_fagec&lang=en]. Last accessed: November 6, 2012.
3. **Istat - Istituto nazionale di statistica. Demography in figures. 2011** [http://demo.istat.it/index_e.html]. Last accessed: November 6, 2012.

4. **Swiss Statistics. Components of population change - Data, indicators.** [http://www.bfs.admin.ch/bfs/portal/en/index/themen/01/06/blank/key/01.html.]. Last accessed: November 6, 2012.
5. Donati S, Baglio G, Spinelli A, Grandolfo ME: **Drug use in pregnancy among Italian women**. *Eur J Clin Pharmacol* 2000, **56**(4):323-328.
6. **Eurostat. Persons with a given education attainment level by sex and age groups (%).** [http://epp.eurostat.ec.europa.eu/portal/page/portal/product_details/dataset?p_product_code=EDAT_LFS_9903.]. Last accessed: November 6, 2012.
7. **Eurostat. Smokers by sex** [http://epp.eurostat.ec.europa.eu/portal/page/portal/product_details/dataset?p_product_code=TPS00169]. Last accessed: November 6, 2012.
8. **The Health and Social Care Information Centre. Statistics on women's smoking status at time of delivery: England, Quarter 1, 2012/13** [http://www.ic.nhs.uk/pubs/wsstd1213q1]. Last accessed: November 12, 2012.
9. De Santis M, De Luca C, Mappa I, Quattrocchi T, Angelo L, Cesari E: **Smoke, alcohol consumption and illicit drug use in an Italian population of pregnant women**. *Eur J Obstet Gynecol Reprod Biol* 2011, **159**(1):106-110.
10. Lemola S, Grob A: **Drinking and smoking in pregnancy: what questions do Swiss physicians ask?** *Swiss medical weekly* 2007, **137**(3-4):66-69.

11. Sayal K, Heron J, Golding J, Alati R, Smith GD, Gray R, Emond A: **Binge pattern of alcohol consumption during pregnancy and childhood mental health outcomes: longitudinal population-based study**. *Pediatrics* 2009, **123**(2):e289-296.

12. **National Institute of Statistics and Economic Studies INSEE. Population** [http://www.insee.fr/en/themes/theme.asp?theme=2]. Last accessed: November 7, 2012.

13. **Statline, Centraal Bureaus voor de Statistiek. Population.** [http://www.cbs.nl/en-GB/menu/themas/bevolking/nieuws/default.htm]. Last accessed: November 10, 2012.

14. **Statistics Austria. Births. Long-term trend and current final annual results. 2011** [http://www.statistik.at/web_en/statistics/population/births/index.html]. Last accessed: November 6, 2012.

15. **STATcube - Statistical Database of Statistics Austria** [http://statcube.at/superweb/login.do?guest=guest]. Last accessed: November 12, 2012.

16. Menai M, Heude B, Slama R, Forhan A, Sahuquillo J, Charles M-A, Yazbeck C: **Association between maternal blood cadmium during pregnancy and birth weight and the risk of fetal growth restriction: The EDEN mother–child cohort study**. *Reprod Toxicol* 2012, **34**(4):622-627.

17. **Jahrbuch der GESUNDHEITSSTATISTIK**. Wein: Statistika Austria. 2011.

18. Leermakers ET, Taal HR, Bakker R, Steegers EA, Hofman A, Jaddoe VW: **A common genetic variant at 15q25 modifies the associations of maternal smoking during pregnancy with fetal growth: the generation R study**. *PloS one* 2012, **7**(4):e34584.

19. De Chazeron I, Llorca P-M, Ughetto S, Vendittelli F, Boussiron D, Sapin V, Coudore F, Lemery D: **Is Pregnancy the Time to Change Alcohol Consumption Habits in France?** *Alcohol Clin Exp Res* 2008, **32**(5):868-873.

20. **Norwegian Institute of Public Health. Medisinsk fødselsregisters statistikkbank - statistikk om alle fødsler i Norge** [http://mfr-nesstar.uib.no/mfr/]. Last accessed: November 12, 2012.

21. Heino A, Gissler M: **Nordic Perinatal Statistics 2010**. Helsinki, Finland: National Institute for Health and Welfare. 2012.

22. **Liitetaulukot - Bilagetabeller - Appendix Tables.** Helsinki, Finland: National Institute for Health and Welfare. 2010.

23. Ystrom E, Vollrath ME, Nordeng H: **Effects of personality on use of medications, alcohol, and cigarettes during pregnancy**. *Eur J Clin Pharmacol* 2012, **68**(5):845-851.

24. Nilsen PER, Holmqvist M, Hultgren EVA, Bendtsen P, Cedergren M: **Alcohol use before and during pregnancy and factors influencing change among Swedish women**. *Acta Obstet Gynecol Scand* 2008, **87**(7):768-774.

25. **Statistics Iceland. Births and Deaths** [http://www.statice.is/Statistics/Population/Births-and-deaths]. Last accessed: November 6, 2012.

26. **The Demographic Yearbook of Russia - statistical handbook**. Moscow: Federal State Statistics Service (Rosstat). 2010.

27. **Demographic Yearbook of Poland**. Warsaw: Central Statistical Office of Poland. 2011.

28. Centers for Disease Control and Prevention: **Current tobacco use and secondhand smoke exposure among women of reproductive age - 14 countries, 2008-2010**. *MMWR Morb Mortal Wkly Rep* 2012, **61**:877-882.

29. Przewoźniak K, W Z: **Ekspertyza „zachowania zdrowotne kobiet w ciąży”**. Warszawa: Zakład Epidemiologii i Prewencji Nowotworów, Centrum Onkologii – Instytut w Warszawie. Posiedzenie Plenarne Rady Naukowej przy Ministrze Zdrowia, Ministerstwo Zdrowia; 2010

30. Kelmanson I, Erman L, Litvina S: **Maternal smoking during pregnancy and behavioural characteristics in 2 - 4-month-old infants.** *Klin Padiatr* 2002, **214**(6):359-364.

31. Hugg T, Jaakkola M, Ruotsalainen R, Pushkarev V, Jaakkola J: **Parental smoking behaviour and effects of tobacco smoke on children's health in Finland and Russia**. *Eur J Public Health* 2008, **18**(1):55-62.

32. Kristjanson AF, Wilsnack SC, Zvartau E, Tsoy M, Novikov B: **Alcohol Use in Pregnant and Nonpregnant Russian Women**. *Alcohol Clin Exp Res* 2007, **31**(2):299-307.

33. Wojtyła A, Kapka-Skrzypczak L, Diatczyk J, Fronczak A, Paprzycki P: **Alcohol-related Developmental Origin of Adult Health--population studies in Poland among mothers and newborns (2010-2012)**. *Ann Agric Environ Med* 2012, **19**(3)

34. **Statistical Yearbook of the Republic of Croatia**. Zagreb: Croatian Bureau of Statistics; 2011

35. **Statistical Yearbook of the Republic of Serbia**. Belgarde: Statistical Office of the Republic of Serbia. 2011.

36. **Statistical Office of the Republic of Slovenia. SI-STAT Data Portal. Population** [http://pxweb.stat.si/pxweb/Database/Demographics/Demographics.asp]. Last accessed: November 7, 2012.

37. Krstev S, Marinković J, Simić S, Kocev N, Bondy S: **Prevalence and predictors of smoking and quitting during pregnancy in Serbia: results of a nationally representative survey**. *Int J Public Health* 2012, **57**(6):875-883.

38. **Statistical Office of the Republic of Serbia. Women and Men in the Republic of Serbia**. Belgrade: Statistical Office of the Republic of Serbia. 2011.

39. Strinic T, Bukovic D, Sumilin L, Radic A, Hauptman D, Klobucar A: **Socio-demographic characteristics and lifestyle habits of pregnant women smokers**. *Coll Antropol* 2005, **29**(2):611-614.

40. Odalovic M, Vezmar Kovacevic S, Ilic K, Sabo A, Tasic L: **Drug use before and during pregnancy in Serbia**. *Int J Clin Pharm* 2012, **34**(5):719-727.

41. Antolic B, Gersak K, Verdenik I, Novak-Antolic Z: **Adverse effects of thyroid dysfunction on pregnancy and pregnancy outcome: epidemiologic study in Slovenia**. *J Matern Fetal Neonatal Med* 2006, **19**(10):651-654.

42. Petković G, Barišić I: **FAS prevalence in a sample of urban schoolchildren in Croatia**. *Reprod Toxicol* 2010, **29**(2):237-241.

43. Hamilton B, Martin J, Ventura S: **Births: Preliminary data for 2010.** *National vital statistics reports.* vol. 60 no 2. Hyattsville, MD: National Center for Health Statistics; 2011

44. **Statistics Canada. Births** [http://www.statcan.gc.ca/daily-quotidien/111220/dq111220g-eng.htm]. Last accessed: November 12, 2012.

45. **Australian Bureau of Statistics. Births, Australia, 2011** [http://www.abs.gov.au/AUSSTATS/abs@.nsf/mf/3301.0]. Last accessed: November 13, 2012.

46. **Panorama de la société 2011 - Les indicateurs sociaux de l'OCDE** [www.oecd.org/els/social/indicateurs/SAG]. Last accessed: November 12, 2012.

47. **United States Census Bureau. The 2012 Statistical Abstract.The National Data Book. Women With Births in the Past 12 Months by Citizenship Status, Educational Attainment, and Poverty Status, by State: 2009** [http://www.census.gov/compendia/statab/cats/births_deaths_marriages_divorces/births.html.]. Last accessed: November 12, 2012.

48. **Canadian Perinatal Health Report, 2008 Edition**. Ottawa: Public Health Agency of Canada. 2008.

49. Sawicki E, Stewart K, Wong S, Leung L, Paul E, George J: **Medication use for chronic health conditions by pregnant women attending an Australian maternity hospital**. *Aust N Z J Obstet Gynaecol* 2011, **51 (4)**:333-338.

50. Pink B: **Australian Social Trends September 2012**. Canberra: Australian Bureau of Statistics. 2012.

51. **Results from the 2010 National Survey on Drug Use and Health: Summary of National Findings**. NSDUH Series H-41, HHS Publication No. (SMA) 11-46 58. Rockville, MD: Substance Abuse and Mental Health Services Administration. 2011.

52. **What Mothers Say: The Canadian Maternity Experiences Survey** Ottawa: Public Health Agency of Canada. 2009.

53. Hotham E, Ali R, White J, Robinson J: **Pregnancy-related changes in tobacco, alcohol and cannabis use reported by antenatal patients at two public hospitals in South Australia**. *Aust N Z J Obstet Gynaecol* 2008, **48**(3):248-254.

54. **Health, United States, 2011**. Hyattsville, MD: With Special Feature on Socioeconomic Status and Health. 2012.

55. Li Z, McNally L, Hilder L, Sullivan E: **Australia's mothers and babies 2009 Perinatal statistics series no. 25. Cat. no. PER 52.** Sydney: AIHW National Perinatal Epidemiology and Statistics Unit.

56. Maloney E, Hutchinson D, Burns L, Mattick RP, Black E: **Prevalence and Predictors of Alcohol Use in Pregnancy and Breastfeeding Among Australian Women**. *Birth* 2011, **38**(1):3-9.
