# Supplementary material for: Herbal medicine use in pregnancy: results of a multinational study
Source: BMC Complement Altern Med. 2013 Dec 12;13:355. doi: 10.1186/1472-6882-13-355 (PMC4029224; doi:10.1186/1472-6882-13-355)
Supplement: Additional file 3 — Medication use in pregnancy with focus on attitudes and perception of risk. This document contains the relevant sections of the questionnaire. [file 1472-6882-13-355-S3.pdf]

Internet questionnaire

Herbal Medicine use in pregnancy: results of a multinational study

A sub-study under the
Multinational Medication Use in Pregnancy Study

INFORMATION ABOUT YOURSELF

1. In which country do you live? **In which region/province do you live?**

Country:________________ **Region: ________________**

2. Are you pregnant right now?

□ Yes □ No

**(If yes in Q2) In which pregnancy week are you?**<br>**From 1 to 44**	**(If No in Q2) How old is your newborn child (in weeks)?**<br>**0-4 / 5-8 / 9-12 / 13-16 / 17-20 / 21-24 / 25-28 / > 29**

3. How many children do you already have from before?

- □ None
- □ One
- □ Two
- □ More than two

4. What is your marital status?

- □ Married
- □ Cohabitant
- □ Single
- □ Divorced/Separated
- □ Other

5. What is the highest education you have completed?

- □ Primary school (8-9 years of education)
- □ High-school (11-13 years of education)
- □ University
- □ Other education

6. What was your work situation when you became pregnant?

- □ Student
- □ Housewife
- □ Health care personnel, i.e., physician, nurse, or pharmacist
- □ Employed in another sector
- □ Job seeker
- □ None of the above

7. Your age: years

INFORMATION ABOUT YOUR PREGNANCY

8. Have you taken folic acid? (alone or as part of multivitamins)

□ Yes, before pregnancy
□ Yes, before and during pregnancy
□ Yes, only during pregnancy
□ No
□ cannot remember

9. Did you smoke cigarettes before becoming pregnant?

□ Yes, regularly
□ Yes, occasionally
□ No, never

(If yes in Q14 as regularly/occasionally) Do you/did you smoke during pregnancy?

□ Yes, more than before
□ Yes, approximately the same
□ Yes, but less
□ No

(If yes) How many cigarettes (on average) do you/did you smoke per day?

□ < 1
□ 1-5
□ 6-10
□ > 11

10. Did you drink any alcohol after finding out that you were pregnant?

□ Yes
□ No
□ Cannot remember

(If yes) How much did you drink (in units)?

1 alcohol unit is equivalent to:
one 25ml single measure of whisky (ABV 40%),
or a third of a pint of beer (ABV 5-6%)
or half a standard (175ml) glass of red wine (ABV 12%).

□ More than 1-2 units per week
□ 1-2 units per week
□ 1-4 units per month
□ 1-2 units during the pregnancy
□ Cannot remember

HEALTH DISORDERS AND MEDICATIONS DURING PREGNANCY

11. Have you experienced any of the disorders listed below during this pregnancy?
If you use or have used any medicines in relation to [each health disorder listed] please enter the names of the medicines.
In which weeks of pregnancy have you used them?

**Health disorder**		**Medicine**	**Period of use (pregnancy weeks)**
Nausea	□ Yes<br>□ No	(If Nausea ticked) If you use or have used any medicines in relation to nausea, please enter the names of the medicines	□ week 0-12<br>□ week 13-24<br>□ week 25- delivery
Heartburn or reflux problems	□ Yes<br>□ No	(If Heartburn ticked) If you use or have used any medicines in relation to heartburn or reflux problem, please enter the names of the medicines	□ week 0-12<br>□ week 13-24<br>□ week 25- delivery
Constipation	□ Yes<br>□ No	(If Constipation ticked) If you use or have used any medicines in relation to constipation, please enter the names of the medicines	□ week 0-12<br>□ week 13-24<br>□ week 25- delivery
Common cold	□ Yes<br>□ No	(If common cold ticked If you use or have used any medicines in relation to common cold, please enter the names of the medicines	□ week 0-12<br>□ week 13-24<br>□ week 25- delivery
Urinary tract infections	□ Yes<br>□ No	(If UTI ticked) If you use or have used any medicines in relation to urinary tract infections, please enter the names of the medicines	□ week 0-12<br>□ week 13-24<br>□ week 25- delivery
Other infections	□ Yes<br>□ No	(If other infections ticked) If you use or have used any medicines in relation to other infections, please enter the names of the medicines	□ week 0-12<br>□ week 13-24<br>□ week 25- delivery
Pain in neck or back or pelvic girdle	□ Yes<br>□ No	(If pain ticked) If you use or have used any medicines in relation to pain in neck or back or pelvic girdle, please enter the names of the medicines	□ week 0-12<br>□ week 13-24<br>□ week 25- delivery
Headache	□ Yes<br>□ No	(If headache ticked) If you use or have used any medicines in relation to headache, please enter the names of the medicines	□ week 0-12<br>□ week 13-24<br>□ week 25- delivery
Sleeping problems	□ Yes<br>□ No	(If sleeping problems ticked) If you use or have used any medicines in relation to sleeping problems, please enter the names of the medicines	□ week 0-12<br>□ week 13-24<br>□ week 25- delivery

12. Did you take any herbal preparations during pregnancy (e.g. ginger, Echinacea, valerian, cranberries**)?**

□ Yes □ No □ Cannot remember

(If yes) Please provide the name of all herbal preparations you have taken during pregnancy**.**
(If yes) What was the reason for taking herbal preparations (health disorder, illness)?
(If yes) In which pregnancy weeks did you take herbal preparations?

**Name of herbal preparation used**	**Reason for use (health disorder, illness)**	**Period of use (pregnancy week**
____________<br>____________<br>____________	____________<br>____________<br>____________	□ week 0-12<br>□ week 13-24<br>□ week 25- delivery
____________<br>____________<br>____________	____________<br>____________<br>____________	□ week 0-12<br>□ week 13-24<br>□ week 25- delivery

13. (If you used herbal preparations during pregnancy) Who recommended to you to take herbal preparations during pregnancy? (You may tick more than one answer)

□ My own initiative
□ Family/friends
□ Physician
□ Midwife/Nurse
□ Pharmacy personnel
□ Herbal shop personnel
□ Internet
□ Magazines, media, etc.
□ Other (please specify:____________)

14. Did you use homeopathic products during pregnancy?

□ Yes □ No □ Cannot remember

(If yes in Q22 above) What was the reason for use?

____

MEDICATIONS FOR CHRONIC DISEASES DURING PREGNANCY

If you use or have used medicines for a chronic disease during your pregnancy fill out this part of the questionnaire (I, II, III) and provide some information about those medicines you use daily.

Do you suffer of any chronic disease? □ Yes □ No

(If Yes above) Please indicate whether you suffer of any of the following chronic diseases.

		**If you use or have used medicines for *X* during your pregnancy, please enter the name of the medicines.**	**In which weeks of pregnancy did you use them?**
Asthma	□ Yes<br>□ No	(If Asthma ticked) If you use or have used medicines for asthma during pregnancy, please enter the names of the medicines.	□ week 0-12<br>□ week 13-24<br>□ week 25-delivery
Allergy	□ Yes<br>□ No	(If Allergy ticked) If you use or have used medicines for allergy during pregnancy, please enter the names of the medicines.	□ week 0-12<br>□ week 13-24<br>□ week 25-delivery
Hypothyroidism (low thyroid hormone)	□ Yes<br>□ No	(If Hypothyroidism ticked) If you use or have used medicines for hypothyroidism during pregnancy, please enter the names of the medicines.	□ week 0-12<br>□ week 13-24<br>□ week 25-delivery
Rheumatic disorders (incl. rheumatoid arthritis, psoriatic arthritis)	□ Yes<br>□ No	(If Rheumatic disorders ticked) If you use or have used medicines for rheumatic disorder during pregnancy, please enter the names of the medicines.	□ week 0-12<br>□ week 13-24<br>□ week 25-delivery
Diabetes (type I or II)	□ Yes<br>□ No	(If Diabetes ticked) If you use or have used medicines for diabetes during pregnancy, please enter the names of the medicines.	□ week 0-12<br>□ week 13-24<br>□ week 25-delivery
Epilepsy	□ Yes<br>□ No	(If Epilepsy ticked) If you use or have used medicines for epilepsy during pregnancy, please enter the names of the medicines.	□ week 0-12<br>□ week 13-24<br>□ week 25-delivery
Depression	□ Yes<br>□ No	(If Depression ticked) If you use or have used medicines for depression, please enter the names of the medicines.	□ week 0-12<br>□ week 13-24<br>□ week 25-delivery
Anxiety	□ Yes<br>□ No	(If Anxiety ticked) If you use or have used medicines for anxiety during pregnancy, please enter the names of the medicines.	□ week 0-12<br>□ week 13-24<br>□ week 25-delivery
Cardiovascular diseases (incl. hypertension, high cholesterol, heart diseases)	□ Yes<br>□ No	(If Cardio disease ticked) If you use or have used medicines for cardiovascular diseases during pregnancy, please enter the names of the medicines.	□ week 0-12<br>□ week 13-24<br>□ week 25-delivery
Others<br>(If Others ticked)<br>(Please specify which other disease(s): ___________)	□ Yes<br>□ No	(If Other disease ticked) If you use or have used medicines for your chronic disease during pregnancy, please enter the names of the medicines.	□ week 0-12<br>□ week 13-24<br>□ week 25-delivery

YOUR VIEWS ABOUT MEDICATIONS

We would like to ask you about your personal views about medicines. There are no right or wrong answers. We are interested in your personal views.

15. These are statements other people have made about medicines in general. Please specify how much you agree or disagree with them by ticking where appropriate. (You may only tick once per line)

	Strongly agree	Agree	Uncertain	Disagree	Strongly disagree
Without medicines doctors would be less able to cure people	◦	◦	◦	◦	◦
Doctors use too many medicines	◦	◦	◦	◦	◦
People who take medicines should stop their treatment for a while every now and again	◦	◦	◦	◦	◦
Most medicines are addictive	◦	◦	◦	◦	◦
Natural remedies are safer than medicines	◦	◦	◦	◦	◦
Medicines do more harm than good	◦	◦	◦	◦	◦
Medicines help many people to live better lives	◦	◦	◦	◦	◦
Medicines help many people to live longer	◦	◦	◦	◦	◦
All medicines are poisons	◦	◦	◦	◦	◦
Doctors place too much trust on medicines	◦	◦	◦	◦	◦
If doctors had more time with patients they would prescribe fewer medicines	◦	◦	◦	◦	◦
In most cases the benefits of medicines outweigh the risks	◦	◦	◦	◦	◦

16. Below are some statements about use of medicines in pregnancy.
Please specify how much you agree or disagree with these statements by ticking where appropriate. (*You may only tick once per line*)

	**Strongly agree**	**Agree**	**Uncertain**	**Disagree**	**Strongly disagree**
I have a higher threshold for using medicines when I am pregnant than when I'm not pregnant	◦	◦	◦	◦	◦
Even though I am ill and could have taken medicines, it is better for the foetus that I refrain from using them	◦	◦	◦	◦	◦
Pregnant women should preferably use herbal remedies than conventional medicines	◦	◦	◦	◦	◦

YOUR ASSESSMENT OF PREGNANCY RISKS

17. Among 100 healthy women in a healthy environment, how many do you think will give birth to a child with a birth defect?

____

18. Here below is a list with various medicines, food and other substances.
Please indicate how harmful you think they are for the foetus in a scale from 0 to 10, where 0 corresponds to 'not harmful' and 10 to 'very harmful'.

If you have not heard before about such substance, tick 'unknown substance'.

	**Unknown substance**	**0**	**1**	**2**	**3**	**4**	**5**	**6**	**7**	**8**	**9**	**10**
Paracetamol/	○	○	○	○	○	○	○	○	○	○	○	○
Antibiotics (*e.g. Penicillins*)	○	○	○	○	○	○	○	○	○	○	○	○
Antidepressants	○	○	○	○	○	○	○	○	○	○	○	○
Thalidomide	○	○	○	○	○	○	○	○	○	○	○	○
Swine influenza vaccine	○	○	○	○	○	○	○	○	○	○	○	○
OTC medicines against nausea/travel sickness	○	○	○	○	○	○	○	○	○	○	○	○
Ginger	○	○	○	○	○	○	○	○	○	○	○	○
Cranberries	○	○	○	○	○	○	○	○	○	○	○	○
Blue veined cheese *(e.g. Gorgonzola)*	○	○	○	○	○	○	○	○	○	○	○	○
Eggs	○	○	○	○	○	○	○	○	○	○	○	○
Alcohol during the 1. trimester (*e.g. wine, beer, spirits*)	○	○	○	○	○	○	○	○	○	○	○	○
Smoking (*e.g. cigarettes*)	○	○	○	○	○	○	○	○	○	○	○	○
Dental X-ray	○	○	○	○	○	○	○	○	○	○	○	○
