# Supplementary material for: Herbal medicine use in pregnancy: results of a multinational study
Source: BMC Complement Altern Med. 2013 Dec 12;13:355. doi: 10.1186/1472-6882-13-355 (PMC4029224; doi:10.1186/1472-6882-13-355)
Supplement: Additional file 4 — The ten most common reasons for using herbal medicines and the most frequently used herbs. A table summarizing the most common reasons given for using herbal medicines and associated most frequently used herb(s), overall and by region. [file 1472-6882-13-355-S4.pdf]

Additional file 4: The ten most common reasons for using herbal medicines and the most frequently used herbs.

	**Overall**			**Western Europe**			**Northern Europe**			**Eastern Europe**		
**Reason for use**	**N**	**% ± SE**	**Top herbs**	**N**	**% ± SE**	**Top herbs**	**N**	**% ± SE**	**Top herbs**	**N**	**% ± SE**	**Top herbs**
Cold or flu	620	18.6 ± 0.7	Cranberry, Echinacea, Garlic, Ginger, Raspberry	136	13.9 ± 1.1	Echinacea, Lemon, Eucalyptus, Peppermint, Sambucus	44	12.9 ± 1.8	Echinacea, Ginger, Sambucus, Peppermint, , Primula	400	24.5 ± 1.1	Cranberry, Raspberry, Garlic, Rutin, Chamomile
Nausea	548	16.5 ± 0.6	Ginger, Peppermint, Artichoke, Lemon	224	22.9 ± 1.3	Ginger, Peppermint, Fennel, Lemon, Raspberry	112	32.8 ± 2.5	Ginger	120	7.3 ± 0.6	Ginger, Artichoke, Peppermint
Urinary tract infections	476	14.3 ± 0.6	Cranberry, Dog rose, Rosemary, Seaside centaury, Lovage	75	7.7 ± 0.9	Cranberry	63	18.5 ± 2.1	Cranberry	321	19.6 ± 1.0	Cranberry, Dog rose, Rosemary, Seaside centaury, Lovage
Sedative/sleeping problems	447	13.4 ± 0.6	Valerian, Motherwort, Lemon balm, Chamomile, Peppermint	100	10.2 ± 1.0	Valerian, Passiflora, Chamomile, Lemon balm	6	1.8 ± 0.7	**	317	19.4 ± 1.0	Valerian, Motherwort, Lemon balm, Peppermint, Chamomile
Constipation	304	9.1 ± 0.5	Psyllium, Fiber crops, Senna, Prunes, Flax	136	13.9 ± 1.1	Psyllium, Fiber crops, Senna, Prunes, Aloe vera	26	7.6 ± 1.4	Prunes, Psyllium, Fiber crops, Flax	54	3.3 ± 0.4	Psyllium, Prunes, Senna, Fiber crops, Fennel
Preparation for labour	206	6.2 ± 0.4	Raspberry, Evening primrose, Sage, Nettle, Fennel	119	12.2 ± 1.0	Raspberry, Evening primrose, Sage	34	10.0 ± 1.6	Raspberry, Evening primrose	16	1.0 ± 0.2	Raspberry
Health promotion	117	3.5 ± 0.3	Cranberry, Ginger, Echinacea, Raspberry, Chamomile	31	3.2 ± 0.6	Echinacea, Cranberry, Ginger, Nettle, Lady's Mantle	12	3.5 ± 1.0	Cranberry, Aloe vera	63	3.9 ± 0.5	Cranberry, Ginger, Dog rose, Lemon, Chamomile
Water retention	94	2.8 ± 0.3	Cowberry, Cranberry, Dog rose, St. John's wort, Bidens	3	*	Nettle	1	*	**	87	5.3 ± 0.6	Cowberry, Cranberry, Dog rose, St. John's wort, Bidens
Gastrointestinal disorders, flatulence	89	2.7 ±0.3	Ginger, Peppermint, Chamomile, Fennel, Cranberry	40	4.1 ± 0.6	Ginger, Peppermint, Fennel, Chamomile	3	0.9 ± 0.5	**	27	1.7 ± 0.3	Ginger, Peppermint, Cranberry
Pain conditions	65	*	Peppermint, Lemon balm, Lemon, Cinnamon, Cloves	14	1.4 ± 0.4	**	8	2.3 ± 0.8	Ginger	34	2.1 ± 0.4	Peppermint, Lemon balm, Lemon, Cinnamon, Cloves

SE: Standard error. Standard errors were calculated for all percentages; however, where the SE > 50% the point estimate is not reported and an "*" is used.
Note: The top 5 herbs are presented where more than 2 women used an herbal medicine. ** no one herb was used by more than 2 women

	North America			South America			Australia		
**Reason for use**	**N**	**% ± SE**	**Top herbs**	**N**	**% ± SE**	**Top herbs**	**N**	**% ± SE**	**Top herbs**
Cold or flu	20	10.9 ± 2.3	Echinacea, Ginger, Garlic	13	16.7 ± 4.2	Chamomile	7	6.1 ± 2.2	Echinacea
Nausea	43	23.4 ± 3.1	Ginger, Peppermint	4	5.1 ± 2.5	**	45	39.5 ± 4.6	Ginger, Peppermint
Urinary tract infections	7	3.8 ± 1.4	Cranberry	3	*	**	7	6.1 ± 2.2	Cranberry
Sedative/sleeping problems	14	7.6 ± 2.0	Chamomile, Valerian	9	11.5 ± 3.6	**	1	*	**
Constipation	43	23.4 ± 3.1	Psyllium, Fiber crops	19	24.4 ± 4.9	Psyllium, Senna, Prunes, Fiber crops	26	22.8 ± 3.9	Psyllium
Preparation for labour	20	10.9 ± 2.3	Raspberry, Nettle, Alfalfa	0	-	-	17	14.9 ± 3.3	Raspberry
Health promotion	6	3.3 ± 1.3	Ginger, Raspberry	3	*	**	2	*	**
Water retention	0	-	-	2	*	**	1	*	**
Gastrointestinal disorders, flatulence	9	4.9 ± 1.6	Ginger	9	11.5 ± 3.6	Chamomile, Peppermint	1	*	**
Pain conditions	4	2.2 ± 1.1	**	5	6.4 ± 2.8	**	0	-	**

SE: Standard error. Standard errors were calculated for all percentages; however, where the SE > 50% the point estimate is not reported and an “*” is used.
Note: The top 5 herbs are presented where more than 2 women used an herbal medicine. ** no one herb was used by more than 2 women
