# Supplementary material for: Herbal medicine use in pregnancy: results of a multinational study
Source: BMC Complement Altern Med. 2013 Dec 12;13:355. doi: 10.1186/1472-6882-13-355 (PMC4029224; doi:10.1186/1472-6882-13-355)
Supplement: Additional file 5 — Factors associated with herbal medicine use, overall and by region. A table summarizing the maternal socio-demographic and lifestyle characteristics of herbal users in this study. This information is presented overall and by region. [file 1472-6882-13-355-S5.pdf]

Additional file 5: Factors associated with herbal medicine use, overall and by region

	Overall			Western Europe			Northern Europe			Eastern Europe		
	N*	(%)	aOR (95% CI)	N	(%)	aOR (95% CI)	N	%	aOR (95% CI)	N	%	aOR (95% CI)
**Region of residence**	-	-	-	842	(26.8)	Reference	305	(11.0)	**0.34 (0.30 - 0.40)**	1,202	(52.0)	**2.97 (2.62 - 3.36)**
**Maternal age (years)**												
<= 20	69	(23.0)	0.76 (0.56 - 1.05)	10	(16.4)	**0.39 (0.19 - 0.82)**	16	(16.7)	1.40 (0.74 - 2.62)	56	(62.9)	**1.68 (1.00 - 2.74)**
21 - 30	1,515	(29.6)	Reference	371	(26.8)	Reference	197	(11.8)	Reference	846	(55.3)	Reference
31 - 40	1,001	(27.0)	0.94 (0.85 - 1.05)	481	(29.1)	1.16 (0.98 - 1.38)	120	(11.9)	1.00 (0.77 - 1.30)	302	(43.3)	**0.65 (0.54 - 0.79)**
>= 40	21	(12.7)	**0.34 (0.21 - 0.55)**	26	(25.0)	0.92 (0.57 - 1.48)	2	(4.9)	0.38 (0.09 - 1.61)	9	(36.0)	0.48 (0.20 - 1.14)
**Previous children**												
No	1,4832	(30.9)	Reference	539	(30.7)	Reference	151	(11.3)	Reference	668	(56.8)	Reference
Yes	1,174	(25.2)	**0.78 (0.71 - 0.87)**	349	(24.1)	**0.66 (0.56 - 0.79)**	184	(12.4)	1.15 (0.90 - 1.47)	545	(46.7)	**0.77 (0.65 - 0.92)**
**Marital Status**												
Married/cohabitating	2,470	(28.3)	Reference	843	(27.7)	Reference	318	(11.8)	Reference	1,134	(51.3)	Reference
Single/divorced/other	136	(24.2)	0.93 (0.75 - 1.17)	45	(28.3)	1.15 (0.79 - 1.67)	17	(13.1)	1.12 (0.65 - 1.95)	79	(60.3)	**1.59 (1.08 - 2.35)**
**Working status**												
Employed but not as HCP	1,664	(30.1)	Reference	542	(26.7)	Reference	194	(12.7)	Reference	791	(53.3)	Reference
Student	267	(32.0)	**1.39 (1.16 - 1.67)**	36	(36.0)	**2.03 (1.28 - 3.22)**	60	(16.2)	1.31 (0.94 - 1.84)	156	(60.2)	1.11 (0.84 - 1.48)
Housewife	219	(26.8)	1.00 (0.83 - 1.21)	101	(28.2)	1.34 (1.02 - 1.75)	19	(14.0)	1.10 (0.65 - 1.86)	75	(46.0)	0.86 (0.61 - 1.21)
HCP	282	(22.8)	**0.80 (0.69 - 0.94)**	121	(32.0)	1.26 (0.99 - 1.60)	37	(7.0)	**0.51 (0.35 - 0.74)**	102	(45.7)	0.75 (0.56 - 1.00)
Job seeker	77	(19.0)	**0.64 (0.49 - 0.85)**	43	(28.7)	1.31 (0.90 - 1.91)	11	(8.7)	0.66 (0.35 - 1.27)	38	(38.8)	**0.58 (0.37 - 0.90)**
Other than above	97	(21.4)	**0.72 (0.59 - 0.92)**	43	(24.4)	0.96 (0.66 - 1.38)	14	(10.4)	0.80 (0.45 - 1.40)	51	(44.0)	0.70 (0.47 - 1.04)
**Education level**												
High school	576	(21.8)	Reference	233	(23.1)	Reference	99	(11.7)	Reference	230	(44.1)	Reference
< High school	68	(16.1)	1.08 (0.81 - 1.46)	52	(32.1)	**1.67 (1.15 - 2.43)**	23	(13.6)	1.15 (0.68 - 1.96)	14	(37.8)	0.81 (0.39 - 1.68)
> High school	1,649	(31.9)	**1.29 (1.14 - 1.46)**	465	(30.0)	**1.26 (1.04 - 1.52)**	163	(11.1)	0.96 (0.73 - 1.28)	862	(53.4)	**1.41 (1.13 - 1.75)**
Other, unspecified	313	(29.7)	**1.60 (1.34 - 1.90)**	138	(28.8)	**1.34 (1.04 - 1.73)**	50	(15.2)	1.45 (1.00 - 2.12)	107	(62.6)	**2.01 (1.39 - 2.90)**
**Folic acid use before and/or during pregnancy**												
Yes	2,503	(29.5)	Reference	851	(30.6)	Reference	311	(12.5)	Reference	1,153	(52.7)	Reference
No	103	(13.0)	**0.43 (0.34 - 0.54)**	37	(33.9)	**0.46 (0.32 - 0.67)**	24	(7.3)	**0.52 (0.34 - 0.81)**	60	(39.0)	**0.63 (0.44 - 0.89)**
**Alcohol use after awareness of pregnancy**												
No	2,018	(26.1)	Reference	663	(26.5)	Reference	298	(11.4)	Reference	935	(50.4)	Reference
Yes	566	(38.2)	**1.40 (1.23 - 1.59)**	213	(31.6)	**1.26 (1.04 - 1.52)**	36	(17.9)	**1.85 (1.25 - 2.73)**	255	(57.4)	**1.33 (1.07 - 1.65)**
Cannot remember	22	(39.3)	1.45 (0.81 - 2.61)	11	(45.8)	**2.46 (1.07 - 5.61)**	1	(14.3)	0.91 (0.11 - 7.79)	22	(57.9)	1.35 (0.69 - 2.64)
**Smoking during pregnancy**												
No	2,435	(93.4)	Reference	832	(30.5)	Reference	314	(11.5)	Reference	1,103	(40.4)	Reference
Yes	171	(6.6)	**0.52 (0.43 - 0.63)**	53	(1.9)	**0.63 (0.46 - 0.87)**	21	(0.8)	0.79 (0.48 - 1.30)	107	(3.9)	**0.52 (0.39 - 0.68)**

Note: * a filter was applied to the Overall Odds Ratio calculation to ensure a homogeneous model resulting in 175 women removed from the calculation. There was no variable included in the model where missing data was > 0.5%.

	North America			South America			Australia		
	N	(%)	aOR (95% CI)	N	(%)	aOR (95% CI)	N	(%)	aOR (95% CI)
**Region of residence**	132	(25.3)	0.95 (0.77 - 1.19)	33	(10.5)	**0.34 (0.24 - 0.50)**	92	4(43.8)	**2.18 (1.63 - 2.92)**
**Maternal age (years)**									
<= 20	8	(21.1)	1.39 (0.53 - 3.63)	3	(8.6)	0.65 (0.18 - 2.43)	2	(20.0)	0.38 (0.06 - 2.52)
21 - 30	66	(22.9)	Reference	33	(15.6)	Reference	42	(44.7)	Reference
31 - 40	64	(32.3)	1.31 (0.84 - 2.05)	23	(25.0)	1.66 (0.87 - 3.18)	46	(44.7)	0.78 (0.40 - 1.51)
>= 40	4	(44.4)	2.26 (0.54 - 9.35)	3	(42.9)	10.48 (1.52 -72.22)	5	(50.0)	1.10 (0.25 - 4.76)
**Previous children**									
No	66	(28.0)	Reference	14	(13.2)	Reference	45	(43.3)	Reference
Yes	76	(25.6)	0.88 (0.57 - 1.37)	48	(20.0)	1.35 (0.67 - 2.72)	50	(44.2)	1.04 (0.53 - 2.03)
**Marital Status**									
Married/cohabitating	125	(28.3)	Reference	49	(17.9)	Reference	90	(46.6)	Reference
Single/divorced/other	17	(18.7)	0.94 (0.48 - 1.84)	13	(17.8)	1.15 (0.55 - 2.40)	5	(20.8)	0.69 (0.20 - 2.36)
**Working status**									
Employed but not as HCP	73	(28.9)	Reference	39	(22.0)	Reference	61	(47.7)	Reference
Student	19	(32.2)	1.55 (0.79 - 3.04)	11	(18.6)	1.04 (0.47 - 2.40)	3	(33.3)	0.64 (0.13 - 3.13)
Housewife	20	(24.4)	0.97 (0.52 - 1.82)	5	(8.8)	0.47 (0.16 - 1.37)	18	(43.9)	0.96 (0.41 - 2.27)
HCP	21	(25.9)	0.79 (0.44 - 1.43)	2	(10.0)	0.26 (0.05 - 1.43)	11	(40.7)	0.60 (0.25 - 1.45)
Job seeker	4	(11.8)	0.50 (0.16 - 1.60)	2	(15.4)	1.11 (0.21 - 5.74)	0	(0.0)	-
Other than above	5	(20.8)	0.79 (0.27 - 2.30)	3	(15.0)	0.70 (0.18 - 2.77)	2	(28.6)	0.61 (0.10 - 3.73)
**Education level**									
High school	20	(15.0)	Reference	25	(18.0)	Reference	19	(30.2)	Reference
< High school	2	(18.2)	1.65 (0.31 - 8.77)	8	(10.7)	0.75 (0.31 - 1.84)	0	(0.0)	
> High school	111	(32.3)	1.96 (1.05 - 3.68)	20	(19.0)	1.04 (0.51 - 2.14)	67	(48.9)	1.62 (0.78 - 3.35)
Other, unspecified	9	(20.0)	1.25 (0.48 - 3.22)	9	(33.3)	2.52 (0.94 - 6.74)	9	(56.3)	3.32 (0.99 - 11.11)
**Folic acid use before and/or during pregnancy**									
Yes	138	(28.8)	Reference	61	(19.4)	Reference	92	(45.5)	Reference
No	4	(7.4)	**0.28 (0.09 - 0.82)**	1	(3.2)	0.18 (0.02 - 1.45)	3	(23.1)	0.61 (0.13 - 2.74)
**Alcohol use after awareness of pregnancy**									
No	102	(23.2)	Reference	56	(19.2)	Reference	67	(42.9)	Reference
Yes	40	(44.4)	**2.25 (1.37 - 3.70)**	5	(11.4)	0.69 (0.24 - 2.00)	28	(47.5)	1.15 (0.59 - 2.25)
Cannot remember	0	(0.0)	-	1	(11.1)	0.80 (0.09 - 7.00)	0	(0.0)	-
**Smoking during pregnancy**									
No	128	(4.7)	Reference	58	(2.1)	Reference	88	(3.2)	Reference
Yes	14	(0.5)	0.96 (0.48 - 1.91)	3	(0.1)	0.56 (0.14 - 2.12)	7	(0.3)	0.36 (0.12 - 1.00)

Note: * a filter was applied to the Overall Odds Ratio calculation to ensure a homogeneous model resulting in 175 women removed from the calculation. There was no variable included in the model where missing data was > 0.5%.
