# Supplementary material for: Herbal medicine use in pregnancy: results of a multinational study
Source: BMC Complement Altern Med. 2013 Dec 12;13:355. doi: 10.1186/1472-6882-13-355 (PMC4029224; doi:10.1186/1472-6882-13-355)
Supplement: Additional file 6 — Source of the recommendation to use herbal medicine in pregnancy, by region and country. A table summarizing the information sources for using herbal medicine in pregnancy, by region and country. Expanded details from Table 2. [file 1472-6882-13-355-S6.pdf]

Additional file 6: Source of the recommendation to use herbal medicine in pregnancy, by region and country.

	Total responses	Percentage of women indicating each recommendation source								
		Own initiative	Physician	Family & Friends	Internet	Midwife, nurse	Pharmacy personnel	Magazine or media	Herbal shop	Other**
	N	%*	%*	%*	%*	%*	%*	%*	%*	%*
**Total**	**3,961**	**28.6**	**21.6**	**16.8**	**11.3**	**7.8**	**6.1**	**3.3**	**3.0**	**1.5**
**Western Europe**	1,315	27.6	14.7	18.6	11.3	10.2	7.7	4.0	4.0	2.1
Austria	62	32.3	21.0	11.3	9.7	*	16.1	*	-	*
France	74	29.7	21.6	14.9	9.5	13.5	8.1	2.7	-	-
Italy	305	23.9	27.9	12.5	9.5	4.6	7.9	4.3	9.5	-
Switzerland	405	30.9	17.0	13.1	7.4	10.1	13.8	1.7	3.0	3.0
The Netherlands	9	22.2	33.3	-	*	-	*	*	-	-
United Kingdom	460	26.3	1.5	29.3	16.1	14.6	0.9	5.9	2.4	3.0
**Northern Europe**	548	31.9	8.6	19.2	15.7	9.1	5.3	3.6	5.7	0.9
Finland	79	39.2	7.6	11.4	13.9	13.9	*	*	6.3	*
Iceland	34	44.1	*	17.6	14.7	14.7	*	-	*	-
Norway	387	27.9	10.1	20.9	17.1	7.8	6.5	4.1	5.4	*
Sweden	48	43.8	*	18.8	8.3	8.3	*	*	8.3	*
**Eastern Europe**	657	28.0	34.5	13.3	10.3	4.9	5.6	2.6	0.8	*
Croatia	116	34.5	17.2	12.9	10.3	*	15.5	5.2	*	*
Poland	422	19.4	34.8	16.1	9.7	5.2	10.4	2.8	*	-
Russia	971	30.2	38.1	13.1	8.8	5.8	1.9	2.2	-	-
Serbia	71	26.8	36.6	8.5	12.7	-	14.1	-	*	-
Slovenia	77	39.0	10.4	5.2	29.9	*	*	5.2	5.2	-
**North America**	209	31.6	8.6	18.2	11.5	13.9	2.4	3.3	3.8	*
Canada	84	28.6	11.9	19.0	8.3	10.7	6.0	*	4.8	*
USA	125	33.6	6.4	17.6	13.6	16.0	-	3.2	3.2	*
**South America**	71	22.5	12.7	36.6	8.5	*	*	5.6	9.9	-
**Australia**	161	29.8	9.9	21.1	9.3	9.3	8.1	*	5.6	*

Note: Women were permitted to indicate more than one source for the recommendation to take an herbal medicine. ** Includes sources such as prenatal, yoga class or CAM Practitioner.

* Standard errors were calculated for all percentage; however, where the SE > 50% the point estimate is not reported and an "*" is used.
